# Central and peripheral blood pressures in relation to the triglyceride-glucose index in a Chinese population

**DOI:** 10.1186/s12933-023-02068-z

**Published:** 2024-01-03

**Authors:** Yin-Hua Sun, Nai-Qing Hu, Xian-Yi Huang, Zheng-Xin Liu, Qi-Yan Li, Qing-Lu Li, Li-Hua Li

**Affiliations:** Department of Gerontology, The First Affiliated Hospital of Dali University, Jiashibo Road 32, Dali, 671000 Yunnan Province China

**Keywords:** Triglyceride-glucose index, Blood pressure, Hypertension, Central hemodynamics, Insulin resistance

## Abstract

**Background:**

The triglyceride-glucose (TyG) index has been proposed as a surrogate marker of insulin resistance. However, the relationship between the TyG index and central blood pressure (BP), has not been well studied in adults.

**Methods:**

A total of 715 Chinese adult participants were enrolled in this study. Anthropometric and BP were assessed. The TyG index was calculated as ln[fasting triglycerides(mg/dL) × fasting glucose(mg/dL)/2]. Central BP was measured using SphygmoCor system.

**Results:**

The participants were stratified into three groups based on the TyG index, and significant differences were observed in metabolic and cardiovascular parameters and the prevalence of hypertension among the groups. Both brachial (β = 1.38, P = 0.0310; group highest vs. lowest, β = 2.66, P = 0.0084) and aortic (β = 2.38, P = 0.0002; group highest vs. lowest, β = 3.96, P = 0.0001) diastolic BP were significantly and independently associated with the TyG index and increasing TyG index tertile. However, there was no independent association between the TyG index and systolic BP. A one-unit increase in the TyG index was associated with a 46% higher risk of hypertension (P = 0.0121), and compared with the lowest group, participants in the highest group had a 95% higher risk of hypertension (P = 0.0057).

**Conclusions:**

Our study demonstrates a significant and independent association between the TyG index and both brachial and aortic diastolic BP in Chinese adults. Furthermore, the TyG index was found to be an independent predictor of hypertension.

## Background

Hypertension, is a significant global health concern that affects more than one billion adults worldwide [1]. It is well established that hypertension is a risk factor for cardiovascular diseases (CVDs), stroke, and renal disease [1]. Primary hypertension in adults is primarily caused by a sedentary lifestyle and subsequent weight gain, as well as insulin resistance which plays a crucial role in its pathogenesis [2, 3]. Recent studies, but not all [4], have shown that the triglyceride-glucose index (TyG index), a measure of insulin resistance, is strongly associated with an increased risk of prehypertension and hypertension in children and adolescents [5], or in middle-aged and elderly adults [6–12], highlighting the importance of early identification of insulin resistance.

Previous studies explored the association between the TyG Index and presence, or incidence of hypertension had produced inconsistent results [5–11]. Additionally, some previous studies have focused solely on specific populations, such as apparently healthy children and adolescents [5], normal-weight Chinese adults [13], or metabolically obese normal-weight subjects [4], hypertensive patients [12], or elderly individuals [11]. Furthermore, all the previous studies have focused on the relationship between the TyG Index and peripheral pressure-based hypertension. Emerging research suggests that central pressure might provide additional predictive value for cardiovascular events over and above peripheral blood pressure (BP) [14]. Central pressure reflects the load on the heart and arteries more accurately, making it a valuable predictor of cardiovascular risk. Only one study, conducted on Chinese hypertensive adults, has found a positive and independent association between the TyG index and central systolic BP [12]. In the present cross-sectional analysis, we investigated associations of the TyG index with central and peripheral BP in a Chinese general population.

## Methods

### Study population

The present cross-sectional analysis was based on the data from an ongoing population study on multiple cardiovascular risk factors in Dali, Yunnan Province, China [15, 16]. The study participants were recruited from two communities in Dali. From October to December 2018, we invited all inhabitants aged 18 years or older to participate. Of those invited, 764 (70%) participated. The ethics committee of the Dali University approved the study protocol. All participants provided written informed consent. We excluded 49 participants because they did not have blood samples collected (n = 6) or arterial (n = 38) or anthropometric measurements (n = 5). Thus, a total of 715 participants were included in the present analysis.

### Fieldwork

Two experienced physicians conducted five consecutive brachial BP measurements for each participant using a mercury sphygmomanometer. Participants were required to rest for a minimum of 5 min in a seated position before these measurements were taken. The average of these five BP readings was used for the analysis. Additionally, the same physicians administered a standardized questionnaire to gather information about participants’ medical history, smoking habits, alcohol consumption, and medication usage. Hypertension was defined as having a brachial systolic BP of at least 140 mmHg or a diastolic BP of 90 mmHg, or if participants were using antihypertensive medications [17].

Anthropometric measurements were conducted by a trained physician, and the body mass index (BMI) was determined by dividing an individual’s body weight in kilograms by the square of their height in meters. Waist circumference measurements were taken at the midpoint between the last rib and iliac crest, while hip circumference measurements were obtained at the widest part of the hip. The waist-to-hip ratio was then calculated by dividing the waist circumference by the hip circumference.

Venous blood samples were collected from participants after an overnight fast to measure various biochemical markers, including plasma glucose, hemoglobin A1c, serum creatinine, and lipid profile. Plasma glucose, serum total cholesterol, triglyceride, high-density lipoprotein cholesterol (HDL-C), low-density lipoprotein cholesterol (LDL-C), and creatinine levels are all tested using standard methods in the laboratory of the First Affiliated Hospital of Dali University. Diabetes mellitus was diagnosed if the fasting plasma glucose level was at least 7.0 mmol/L, or if the hemoglobin A1c level was at least 6.5%, or if individuals were using antidiabetic agents [18]. TyG index TyG = Ln [fasting triglycerides (mg/dL)*fasting plasma glucose (mg/dL)/2] [19].

### Central BP measurement

To ensure stability during measurements, a qualified physician conducted all arterial assessments using applanation tonometry after participants had rested in a supine position for 15 min. Participants were instructed to abstain from smoking, intense physical activity, and the consumption of alcohol or caffeine-containing beverages for a minimum of 2 h before the examination. For recording arterial waveforms, we utilized a high-fidelity SPC-301 micromanometer manufactured by Millar Instruments, which was connected to a laptop computer running SphygmoCor version 7.1 software developed by AtCor Medical. To maintain data quality, recordings were excluded if consecutive waveform variations exceeded 5% or if the pulse wave signal amplitude fell below 80 mV. Prior to the SphygmoCor recordings, pulse wave calibration was performed using the average of two consecutive brachial BP readings measured in the supine position. This calibration was executed with a validated Omron HEM-7051 oscillometric BP monitor manufactured by Omron. Using the radial signal as input, the SphygmoCor software applied a validated generalized transfer function to compute the aortic pulse wave [20]. Subsequently, central systolic and diastolic BP values were derived from the aortic pulse wave.

### Statistical analysis

Database management and statistical analyses were performed using SAS version 9.4 and EmpowerStats version 4.1. Means and proportions were compared with the analysis of variance, chi-square test, or Fisher’s exact test, respectively. The TyG index was used as both a continuous variable and a categorical variable (tertiles of the TyG index) to analyze its relationship with peripheral and central BP. We performed single (model 1) and multiple (model 2) linear regression analyses to study the associations of the TyG index with central and peripheral BP. The variables adjusted in the model 2 included sex, age, BMI, current smoking, current drinking, and current antihypertensive treatment. We performed single and multiple logistic regression analyses to study the associations of the TyG index with risk of hypertension. P values < 0.05 were considered statistically significant.

## Results

### Characteristics of the study population

The 715 participants included 467 (65.31%) women, 226 (31.61%) hypertensive patients. Table 1 displays the clinical and laboratory characteristics of the study cohort. The participants were categorized into three groups based on their TyG index levels. Substantial differences in metabolic parameters and BP were evident across these groups. Variables such as BMI, waist circumference, hip circumference, waist-to-hip ratio, total cholesterol, triglyceride, brachial BP, pulse rate, and aortic BP exhibited positive associations with the increasing TyG index tertile. Furthermore, the TyG index tertile showed positive associations with current smoking, current drinking, and the prevalence of diabetes mellitus, hypertension, and ongoing antihypertensive treatment. It’s worth noting that there were fewer women in the second and third tertile of the TyG index groups.

**Table 1:** Basic characteristics of the study population according to the TyG Index

Variables	T1 (< 8.54)	T2 (8.54-9.00)	T3 (>9.0)	P
N	238	238	239	
Age, years	48.99 ± 14.28	52.49 ± 12.20	54.07 ± 10.49	< 0.001
Body mass index, kg/m^2^	22.30 ± 3.05	24.32 ± 3.14	25.09 ± 3.14	< 0.001
Waist circumference, cm	79.8 ± 8.9	86.4 ± 8.3	88.9 ± 8.5	< 0.001
Waist-to-hip ratio	0.87 ± 0.05	0.90 ± 0.05	0.91 ± 0.05	< 0.001
Total cholesterol, mmol/L	4.44 ± 0.74	4.82 ± 0.85	5.15 ± 1.04	< 0.001
Triglyceride, mmol/L	0.93 (0.78–1.09)	1.52 (1.32–1.71)	2.45 (2.07–3.19)	< 0.001
TyG	8.19 ± 0.26	8.77 ± 0.14	9.53 ± 0.54	< 0.001
LDL-C, mmol/L	2.27 ± 0.54	2.68 ± 0.63	2.74 ± 0.78	< 0.001
HDL-C, mmol/L	1.44 ± 0.25	1.35 ± 0.22	1.38 ± 0.22	< 0.001
Serum creatinine, µmol/L	69.80 ± 16.13	72.48 ± 19.12	73.48 ± 18.93	0.072
Brachial SBP, mmHg	115 ± 19	119 ± 16	124 ± 17	< 0.001
Brachial DBP, mmHg	73 ± 11	78 ± 11	80 ± 11	< 0.001
Brachial PP, mmHg	41 ± 12	41 ± 10	44 ± 12	0.010
Pulse rate, beats/min	71 ± 8	72 ± 9	74 ± 9	0.001
Aortic SBP, mmHg	111 ± 18	116 ± 15	121 ± 16	< 0.001
Aortic DBP, mmHg	74 ± 12	78 ± 11	83 ± 11	< 0.001
Aortic PP, mmHg	36 ± 10	37 ± 8	38 ± 9	0.176
Women, n (%)	180 (75.63%)	155 (65.13%)	132 (55.23%)	< 0.001
Current smoking, n (%)	32 (13.45%)	49 (20.59%)	64 (26.78%)	0.001
Current drinking, n (%)	18 (7.56%)	21 (8.82%)	42 (17.57%)	< 0.001
Diabetes mellitus, n (%)	1 (0.42%)	19 (7.98%)	56 (23.43%)	< 0.001
Hypertension, n (%)	45 (18.91%)	74 (31.09%)	107 (44.77%)	< 0.001
Current antihypertensive treatment, n (%)	28 (11.76%)	47 (19.75%)	64 (26.78%)	< 0.001

T1, T2, and T3 refers to the first Tertile, second Tertile, and third Tertile of TyG index, respectively. The values in the table are presented as mean ± standard deviation, or median (interquartile range), or n (%). TyG index, triglyceride-glucose index; LDL-C, low-density lipoprotein cholesterol; HDL-C, high-density lipoprotein cholesterol; SBP, systolic blood pressure; DBP, diastolic blood pressure; PP, pulse pressure

### Linear regression analysis of the TyG index and brachial BP

The association between the TyG index and brachial BP was further examined through both linear single and multiple regression analyses, considering the TyG index as both a continuous and categorical variable (divided into tertiles, with the first tertile as the reference). In the unadjusted model (model 1), the TyG index exhibited an association with elevated brachial systolic and diastolic BP, as well as pulse pressure (P ≤ 0.0139). However, after adjusting for confounding factors, only brachial diastolic BP showed a significant association with a higher TyG index. Specifically, a one-unit increase in the TyG index correlated with a 1.38 mmHg increase in brachial diastolic BP (P = 0.0310). Furthermore, when compared to participants in the lowest TyG index tertile, those in the highest tertile had a 2.66 mmHg higher brachial diastolic BP (P = 0.0084) (Table 2).

**Table 2:** The relationship between the TyG index and brachial blood pressure

Variables	Model 1	Model 2
Brachial SBP, mmHg		
TyG index	6.07 (4.11, 8.02) < 0.0001	0.58 (-1.25, 2.40) 0.5348
TyG Tertile		
T1	0	0
T2	4.05 (0.92, 7.19) 0.0115	-1.24 (-3.99, 1.51) 0.3768
T3	9.49 (6.36, 12.63) < 0.0001	1.36 (-1.51, 4.23) 0.3535
Brachial DBP, mmHg		
TyG index	4.28 (3.05, 5.51) < 0.0001	1.38 (0.13, 2.64) 0.0310
TyG Tertile		
T1	0	0
T2	4.36 (2.39, 6.33) < 0.0001	1.64 (-0.25, 3.53) 0.0893
T3	6.94 (4.98, 8.91) < 0.0001	2.66 (0.69, 4.64) 0.0084
Brachial PP, mmHg		
TyG index	1.79 (0.52, 3.05) 0.0058	-0.80 (-1.99, 0.38) 0.1819
TyG Tertile		
T1	0	0
T2	-0.31 (-2.34, 1.72) 0.7653	-2.88 (-4.65, -1.11) 0.0015
T3	2.55 (0.52, 4.58) 0.0139	-1.30 (-3.15, 0.55) 0.1678

T1, T2, and T3 refers to the first Tertile, second Tertile, and third Tertile of TyG index, respectively. Data are presented as β (95% CI) and P value. Model 1: Unadjusted; Model 2: Adjusted for sex, age, body mass index, current smoking, current drinking, and current antihypertensive treatment. TyG index, triglyceride-glucose index; SBP, systolic blood pressure; DBP, diastolic blood pressure; PP, pulse pressure

#### Linear regression analysis of the TyG index and aortic BP

The association between the TyG index and aortic BP was further examined through both linear single and multiple regression analyses, considering the TyG index as both a continuous and categorical variable (divided into tertiles, with the first tertile as the reference). In the unadjusted model (model 1), the TyG index exhibited an association with elevated aortic systolic and diastolic BP (P ≤ 0.0018). However, after adjusting for confounding factors, only aortic diastolic BP showed a significant association with a higher TyG index. Specifically, a one-unit increase in the TyG index correlated with a 2.38 mmHg increase in aortic diastolic BP (P = 0.0002). Furthermore, when compared to participants in the lowest TyG index tertile, those in the highest tertile had 3.96 mmHg higher aortic diastolic BP (P = 0.0001) (Table 3).

**Table 3:** The relationship between the TyG index and aortic blood pressure

Variables	Model 1	Model 2
Aortic SBP, mmHg		
TyG index	6.68 (4.86, 8.50) < 0.0001	1.36 (-0.38, 3.11) 0.1268
TyG Tertile		
T1	0	0
T2	4.69 (1.76, 7.62) 0.0018	-0.60 (-3.22, 2.03) 0.6564
T3	10.48 (7.56, 13.41) < 0.0001	2.52 (-0.22, 5.27) 0.0718
Aortic DBP, mmHg		
TyG index	5.72 (4.45, 6.98) < 0.0001	2.38 (1.11, 3.65) 0.0002
TyG Tertile		
T1	0	0
T2	4.17 (2.13, 6.20) < 0.0001	1.00 (-0.91, 2.91) 0.3039
T3	9.00 (6.96, 11.03) < 0.0001	3.96 (1.97, 5.95) 0.0001
Aortic PP, mmHg		
TyG index	0.97 (-0.06, 1.99) 0.0657	-1.03 (-2.05, -0.01) 0.0483
TyG Tertile		
T1	0	0
T2	0.53 (-1.12, 2.18) 0.5294	-1.59 (-3.13, -0.05) 0.0432
T3	1.54 (-0.10, 3.19) 0.0669	-1.39 (-3.00, 0.22) 0.0907

T1, T2, and T3 refers to the first Tertile, second Tertile, and third Tertile of TyG index, respectively. Data are presented as β (95% CI) and P value. Model 1: Unadjusted; Model 2: Adjusted for sex, age, body mass index, current smoking, current drinking, and current antihypertensive treatment. TyG index, triglyceride-glucose index; SBP, systolic blood pressure; DBP, diastolic blood pressure; PP, pulse pressure

### Logistic regression analysis of the TyG index and risk of Hypertension

Both before and after adjusted for confounding factors, higher TyG index was associated with a higher risk of hypertension. Indeed, a one-unit increase of TyG index was associated with a 45% higher risk of hypertension (P = 0.0121) after adjusting for multiple risk factors. Furthermore, when compared to participants in the lowest TyG index tertile, those in the highest tertile had a 95% higher risk of hypertension (P = 0.0057) (Table 4).

**Table 4:** The relationship between TyG index and risk of hypertension

Variables	Model 1	Model 2
TyG index	2.16 (1.67, 2.79) < 0.0001	1.46 (1.09, 1.96) 0.0121
TyG Tertile		
T1	1.0	1.0
T2	1.94 (1.27, 2.96) 0.0023	1.28 (0.79, 2.09) 0.3120
T3	3.48 (2.30, 5.25) < 0.0001	1.95 (1.22, 3.14) 0.0057

T1, T2, and T3 refers to the first Tertile, second Tertile, and third Tertile of TyG index, respectively. Data are presented as odds ratio (95% CI) and P value. Model 1: Unadjusted; Model 2: Adjusted for sex, age, body mass index, current smoking, and current drinking. TyG index, triglyceride-glucose index

## Discussion

In this study, we identified a significant and independent relationship between the TyG index, both brachial and aortic diastolic BP, and the risk of hypertension. As central pressure may be predictive of cardiovascular events, in addition to and independent of brachial pressure, our finding therefore may have clinical implications for cardiovascular prevention by improving insulin sensitivity.

To the best of our knowledge, this is the first study to demonstrate such a relationship between the TyG index and aortic diastolic BP in the general population. Previous studies mainly focused on the association of the TyG index with prevalence or incidence of hypertension [5, 9, 11, 21, 22], while very few studies pay attention to BP components [12] or hypertension subtypes [8]. Our study again confirmed that a higher TyG index was related to an increased risk of hypertension. However, we found that the TyG index is primarily associated with elevated peripheral and central diastolic BP, and its relationship with systolic BP is not independently significant. Therefore, it is inferred that hypertension related to the TyG index is mainly characterized by isolated diastolic hypertension.

The TyG index, which is a surrogate marker of insulin resistance, is known to be associated with metabolic parameters, hypertension [5, 9, 11, 21, 22], and CVDs [23–25]. Several insulin resistance related mechanisms are involved in the pathogenesis of hypertension. Previous studies have shown that insulin resistance can promote the formation of advanced glycation end products, increase oxidative stress and systemic inflammation, lead to mitochondrial dysfunction, dyslipidemia, endothelial dysfunction, decrease nitro oxide synthesis, and increase volume load. These mechanisms together lead to increased peripheral vascular resistance and preload, ultimately resulting in the development of hypertension [26]. Insulin resistance also plays a key role in the progression of hypertension-induced target organ damage, like left ventricular hypertrophy, atherosclerosis and chronic kidney disease [27].

Our study did not find a significant independent association between the TyG index and peripheral or central systolic BP, possibly due to the majority of the study participants being middle-aged with a relatively lower proportion of obesity. In fact, the Reaction Study found that an elevated TyG index is significantly associated with hypertension in the subgroup of the oldest age (≥ 65) (OR 1.67, 95% CI 1.30–2.14, P < 0.0001), as well as with obesity (BMI ≥ 28 kg/m^2^) (OR 1.85, 95% CI 1.29–2.66, P = 0.0009) or lower estimated glomerular filtration rate (eGFR) (< 90 mL/(min·1.73 m^2^)) (OR 1.72, 95% CI 1.33–2.21, P < 0.0001) [11]. We speculate that in older individuals, insulin resistance may more easily lead to arterial stiffness [28, 29] and increased volume load, manifesting mainly as isolated systolic hypertension. However, this study cannot rule out the possibility that antihypertensive, lipid-lowering, and hypoglycemic treatments may attenuate the relationship between the TyG index and systolic BP. Indeed, Wang et al. found a significant association between the TyG index and central systolic BP in 9249 Chinese hypertensive adults, and the association was limited to hypertensive patients who do not use antihypertensive drugs (β = 1.03, 95% CI: 0.46–1.60, P < 0.001) [12]. Furthermore, central diastolic BP was not assessed in their study, and their study population consisted exclusively of hypertensive patients [12]. Therefore, a direct comparison between their findings and ours is not possible.

Our study examined the relationship between peripheral and central BP and the TyG index in a Chinese population, further highlighting the important value of the TyG index in predicting hypertension. However, our study is limited by its cross-sectional design, which precludes any causal inference. Additionally, we only performed a single measurement of plasma glucose and serum triglyceride concentrations, which are influenced by various factors and may fluctuate over time. Furthermore, central BP was measured using a noninvasive device, and our findings may be specific to this device. Nonetheless, the SphygmoCor device has been previously validated for estimating central BP [30]. Additionally, the present study was conducted solely on a Chinese general population. As a result, the generalizability of our findings is constrained.

## Conclusions

In conclusion, our study has demonstrated a significant and independent association between the TyG index and both brachial and aortic diastolic BP. Furthermore, we found that the TyG index is independently associated with an increased risk of hypertension. As a simple-to-calculate marker of insulin resistance, the TyG index appears to be a useful indicator of BP and cardiovascular risk. However, further large-scale prospective studies are needed to fully elucidate the exact mechanism underlying the relationship between the TyG index and hypertension.

## Data Availability

The datasets used during the study are available from the corresponding author (Prof. Li-Hua Li) on reasonable request.

## List of abbreviations

TyG index: Triglyceride-glucose index
BP: Blood pressure
BMI: Body mass index
HDL-C: High-density lipoprotein cholesterol
LDL-C: Low-density lipoprotein cholesterol
CVDs: Cardiovascular diseases
